# The pharmaceutical suitcase trade and the need for multisectoral regulation-unproven COVID-19 (Ivermectin and HCQ remedies unmask an insidious health danger in a Caribbean Island)

**DOI:** 10.3389/fpubh.2024.1353516

**Published:** 2024-12-12

**Authors:** Sandeep Bhupendra Maharaj, Darren Dookeeram, Roger Hosein, Kelvin Ramkissoon, Amrica Ramdass, Darleen Y. Franco, Shalini Pooransingh

**Affiliations:** ^1^Faculty of Medical Sciences, The University of the West Indies St. Augustine, St. Augustine, Trinidad and Tobago; ^2^Faculty of Social Sciences, Department of Economics, The University of the West Indies St. Augustine, St. Augustine, Trinidad and Tobago; ^3^Independent Researcher, Port of Spain, Trinidad and Tobago; ^4^The University of the West Indies St. Augustine, St. Augustine, Trinidad and Tobago; ^5^Department of Family Medicine, North West Regional Health Authority, Port of Spain, Trinidad and Tobago

**Keywords:** suitcase trade, pharmaceutical regulation, health in all policy, public health, Ivermectin

## Abstract

This article seeks to highlight an aspect of the illegal pharmaceutical trade in the Caribbean. With the advent of COVID-19 there has been a shortage of a number of drugs in the formal sector. This is largely due to restrictions on foreign exchange, importation delays and sensationalized reporting of unrecommended drugs having a curative effect on COVID-19 patients. This article examines the issue of “the informal suitcase trading” of these drugs. It posits a need for a collaborative and multi-sectoral approach to mitigate the negative effects of the practice on health, trade and national security.

## Defining the issue

**Figure 1:**
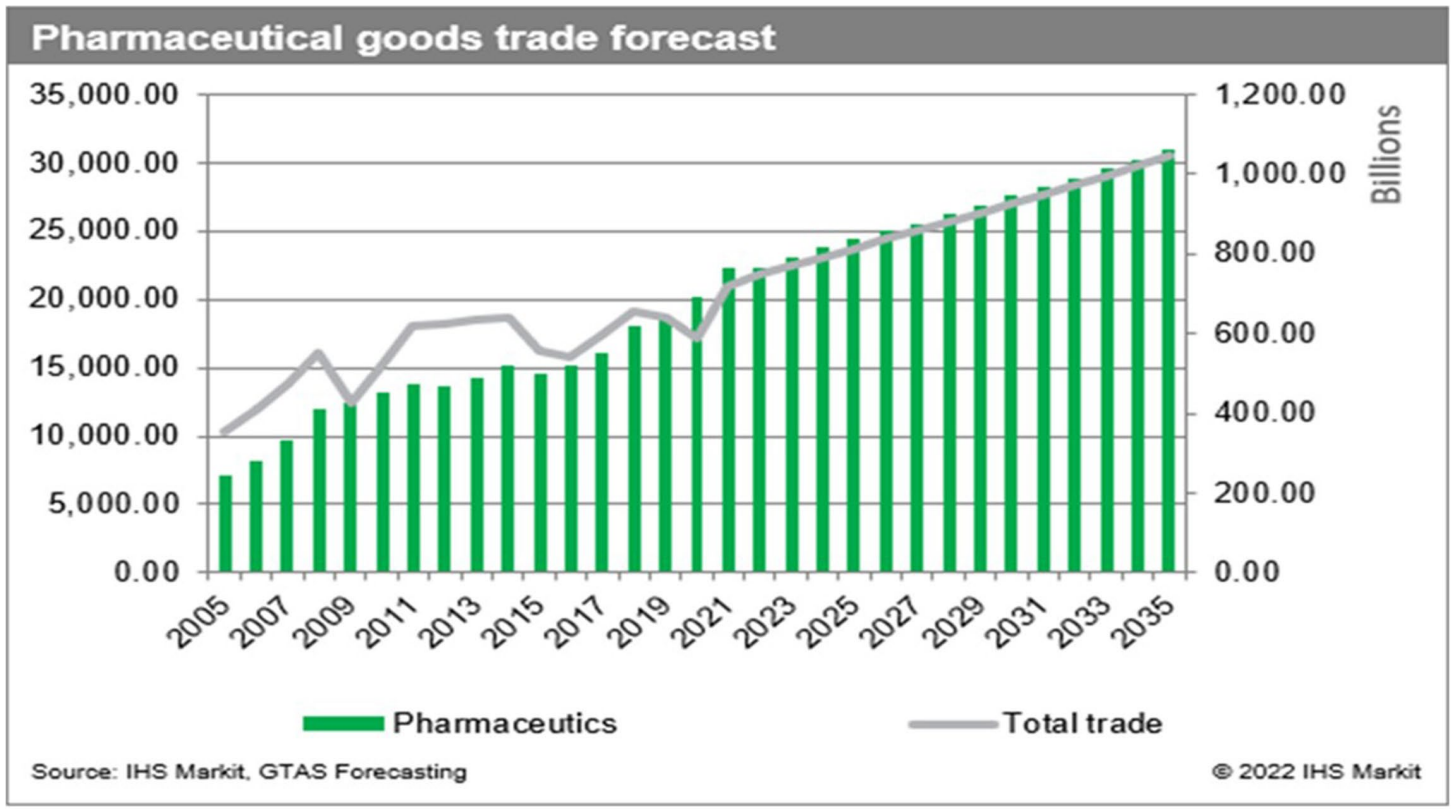
Pharaceutical goods trade forecast 2022.

The volume of international trade in regulated pharmaceuticals has seen a substantial increase over the past two decades, making it one of the most profitable industries in the global economy. This trend is forecasted to grow exponentially making this industry increasingly attractive and lucrative. This is illustrated in Figure 1, taken from the S&P global trade website (1).

## Suitcase trade: the unspoken component of pharmaceuticals

The International Monetary Fund recognizes suitcase trading as “a form of unrecorded or under-recorded international transaction in goods that is currently existent at the edges of formal trade.” Suitcase traders benefit from avoidance of customs duties, tariffs, freight charges and regulatory oversight in the recipient country. The unrecorded items are entered and sold at market or above market prices and also evade imposts. Most importantly, they escape the oversight of regulatory processes (2). Compared to the established trade patterns illustrated in Figure 1, little is known about the volume of trade generated by this informal sector and only rough estimates can be discerned by assessing movement of counterfeit medication. Suitcase trading also includes authentic medications which enter by irregular and non-regulated means.

This form of trade is considered opportunistic since it is dependent on the rigidity of the receiving markets where a demand for the goods, coupled with anomalies in regulatory processes provide informal traders with an unfair economic advantage (2). The suitcase trading of pharmaceuticals takes advantage of loose regulatory and economic loopholes as well as gaps in enforcement. This phenomenon was highlighted in Trinidad and Tobago by the Minister of Health in recent years in the face of Ivermectin during the COVID-19 pandemic (3) but has been long recognized in the literature due to its potentiation of counterfeiting (4), illicit use (5) and illegal trade disruption (6). This issue came surging to the fore again in 2023 with news outlets reporting police exercises that seized hundreds of thousands of United States dollars (USD) worth of unregistered pharmaceuticals from the bonds and warehouses in Trinidad and Tobago (7, 8). From the health perspective, the use of unregistered medication poses a potential risk to patient safety since its effect may be unpredictable and even unsafe. Health systems therefore require a robust public health education thrust to sensitize potential buyers to the risks (9). Unregistered medication also results in a range of non-health effects extending to several sectors including trade and national security. A multi-sectoral approach is therefore required for mitigation (10).

## Root causes of the problem

Ayduin et al. in a regional analysis of the economic impact of the suitcase trade on formal trade explore several reasons for its existence and propagation. While primarily taken in the context of its impact on foreign trade, the effect on the pharmaceutical industry, and by extension health, is noteworthy (11). The first context considers the suitcase trade as a by-product of global trade in an almost disruptive innovation to normal practices. This speaks to the change in consumption tendencies in populations and the evolution of providers who seek to fill new gaps between supply and demand (12). In the health context, this is manifested as drug shortages where regulated distributors are unable to keep pace with demand. This can result in disproportionate price differentials between regulated and unregulated products. It has also been noted that highly controlled pharmaceutical products potentially propagate unregulated trade making it highly illicit. The recent worldwide scourge of opiate addiction is one such example and has created another dimension of suitcase trade where products have been confiscated on their way from the Global South to the Global North (13, 14). The unregulated trade therefore is not restricted to developing and transition states. It provides a platform for pharmaceuticals to enter the more regulated markets in the metropolitan countries, as recently seen in the arrest and detention of two visiting British nationals for possession of hundreds of bottles of Codeine linctus stored at paid accommodation facilities. It highlights the two-way element of the trade and the entry of restricted pharmaceuticals into states with more advanced capacity and detection mechanisms (15).

The second context considers the evolution of suitcase trading in transition economies where countries with high expenditure on non-market items such as defense can create a gap in supply for routine products which ultimately drives up prices (16). In the health sectors of the Caribbean, halted or disrupted importation may decrease the availability of certain drugs to vulnerable groups. The Caribbean does not historically engage in high expenditures on defense and the regional Gross Domestic Expenditures are considerably less than partners in the Global North. The Island States nevertheless face growing fiscal restraints on health in the post pandemic era. Resultantly, health sectors are subject to declining budgetary allocations for some pharmaceuticals and medical devices leading to decreased supplies and disproportionate pricing in retail markets.

The third context is the cultural dynamic of the suitcase trade where the unregulated suppliers in communities are considered an integral part of the supply chain (17). The rampant and unrestricted influence of social media has had a significant impact on health matters in the Caribbean as was exemplified throughout the pandemic with vaccination (18). In the context of peddled misinformation, this is impactful since unregulated traders can be perceived as a panacea to gaps in health systems.

## COVID-19: Ivermectin and hydroxychloroquine (HCQ)

Ivermectin, a drug traditionally utilized for parasitic infections, came into the spotlight in 2019 as the world sought an effective therapeutic for COVID-19. The Food and Drug Administration of the United States constantly rebutted claims that it was effective and repeatedly underscored that no approvals were granted for its use in the United States or the Caribbean (19, 20). Counterproductively, certain sectors of society held tightly to ill-founded and unscientific information regarding its efficacy which spurned its global demand. In this regard, the stage had been set, with legal barriers to importation and a demand in the local market to foster the suitcase trade of Ivermectin and Hydroxychloroquine (HCQ) in Trinidad and Tobago as demand outstripped market supply (21).

During the COVID-19 pandemic in Trinidad and Tobago, unethical practices in healthcare became rampant. Medications like Ivermectin, not officially available in the country, were smuggled and sold at inflated prices. Hydroxychloroquine, typically prescribed for autoimmune diseases, was hoarded for COVID-19 treatment, leaving regular patients without their necessary medication. A senior physician recounted a case where a colleague, despite having minimal COVID-19 symptoms, was prescribed an unnecessary array of drugs including Intravenous Meropenem, Azithromycin, Hydroxychloroquine, and Ivermectin, none of which were appropriate for treating COVID-19. These actions were fueled by misinformation spread through social and mainstream media, sidelining scientific evidence. The pandemic saw the rise of unqualified individuals presenting themselves as experts, promoting ineffective treatments like high-dose Vitamin C, unnecessary medications and discrediting vaccines. The integrity of journalism in reporting accurate information eroded, while financial motives further compromised healthcare providers’ ethical standards (22).

Based on a newspaper report in 2020, the local lupus advocacy group expressed concerns about the unavailability of the drug. Due to rumors of its benefits in the treatment of COVID-19, pharmacy dealers engaged in price gouging and tripled the price from $3.20TTD to $10TTD.The result was predictable panic buying which led to an exhaustion of stock in the private sector. Patients afflicted with diseases for which HCQ was the recommended treatment, were unable to access it. There were also suspicions of illegal brands on the market which could not be verified (3, 23).

Based on Ayudin’s review article, in the case of high demand for unproven remedies during the COVID-19 pandemic, the clear precipitants are demonstrated where an astronomical increase in demand well outstripped supply which allowed unregulated suppliers who had gained public trust, to flourish. This unregulated supply stream proved costly to buyers and disadvantaged some segments of the population who were unable to afford the drug and may have caused medical complications.

The surge in demand for medications like Hydroxychloroquine, driven by unsubstantiated claims of its effectiveness against the virus led to panic buying and stockpiling, causing shortages that affected patients who rely on the drug for conditions like rheumatoid arthritis and lupus. Global responses to these shortages included regulatory measures such as export bans by the NHS in the UK and restrictions in India to safeguard domestic supply, exacerbating shortages worldwide. Concerns also arose over the availability of active pharmaceutical ingredients (APIs) from China and India, crucial for global drug manufacturing. In Trinidad and Tobago, price hikes and hoarding of medications underscored fears of supply disruptions, particularly for chronic diseases. Regulatory bodies and health officials urged restraint and responsible prescribing to manage these challenges during the pandemic (24).

Hydroxychloroquine, Azithromycin, and Ivermectin gained widespread attention in Latin America and the Caribbean (LAC) during the early days of the pandemic. Social media played a significant role in disseminating misinformation and promoting these unproven treatments, contributing to a broader “infodemic” in the region. Countries like Peru and Costa Rica faced particularly high risks of misinformation spreading, correlating with increased COVID-19 mortality rates. This phenomenon highlighted the challenge of combating false narratives and maintaining adherence to proven public health strategies amidst a global health crisis (25).

## The dangers and threats of the practice

The World Health Organization’s 2022 publication on Therapeutics and COVID-19 recommended against the use of Ivermectin outside of research settings and also strongly against the use of HCQ for any treatment of COVID-19 (26). The result of acquisition of pharmaceuticals other than by regulated and registered mechanisms include the injection of counterfeit medications into markets, encouraging abuse and misuse of certain drug classes, together with the creation of anti-competitive behaviors. These factors are disadvantageous to market providers who operate within the legal remit (2). This is particularly relevant to the pharmacy markets in Trinidad and Tobago where the effects have been documented and placed in the public domain (3). These factors may have had the effect of suppressing growth of small and medium enterprises which act within the legal parameters of the healthcare trade, and by extension, block equity access to service providers who depend on these enterprises for economic activity.

According to the Food and Drug Administration (FDA) of the United States of America, the unregulated procurement of pharmaceuticals in a country, such as through online purchases, significantly increases the likelihood of the entry of counterfeit medications into the market. It notes that this potentially harmful activity should be counter-measured through monitoring and legal consequences for those found in breach of regulations (27). The potentially serious health effects are echoed by the European regulators who cite a wide spectrum of medication categories as being vulnerable to counterfeiting, but notes significant efforts by the European Police Agencies to curb this problem (28). The World Health Organization recognizes the significant global challenges posed by counterfeit medical products and medication and through its Member States Mechanism on Substandard and Falsified Medical Products, provides a forum for convention, coordination and active programs to allow multilateral mechanisms to counter what it calls a “pervasive problem” (29).

The unregulated presence of Ivermectin and Hydroxychloroquine through the suitcase trade in Trinidad & Tobago can pose potentially serious public health risks which include counterfeit pharmaceuticals, drug interactions, adverse drug events and improper handling and disposal of medication. These clinical hazards support the need for strengthening pharmaceutical regulations and empowering professionals in the field (30). This is relevant in a global health environment where even drugs that have been legally imported sometimes fail medical standards with extreme detriment to consumers such as the case of contaminated cough syrups in India in 2023 (31). It is therefore imperative that all medical products and drugs be subject to quality assurance testing; the suitcase trade bypasses this step with potentially devastating effect.

## The legislative landscape

As it stands, there is sufficient legislative teeth to address the scourge of the illicit entry of pharmaceutical drugs into Trinidad and Tobago. There is a wide spectrum of enforcement powers under the Food and Drugs Act and Customs Act of Trinidad and Tobago. The Food and Drugs Act grants the inspectors power to evaluate and examine imported pharmaceuticals at any time (32). The Customs Act confers the power to demand information and seize articles in circumstances where requisite legal protocols are violated (33). There are also like provisions in the Barbados Customs Act (34) and the Jamaica Customs Act (35). With adequate laws in place, the authors posit that the problem appears to be with the enforcement of the law and the absence of a collaborative multi-sectoral approach. With increased capacity of the regulatory bodies, proper training of Customs and Excise Officers and law enforcement agencies in national security, this problem can be effectively addressed and the negative effects mitigated.

## The need for multisectoral regulation

To safeguard the public, there are some multi-sectoral actions that the policy makers and relevant regional bodies can consider.

1. Education – the public, healthcare professionals and officers at ports of entry need to be made aware of the existence of this trade and its potential for grave effects on public health. Public Health Education should continue to be a focus as a mitigation strategy.
2. Legal and regulatory frameworks –The capacity of the Drug Inspectorate needs strengthening to include ready attendance at points of entry and to conduct audits on pharmacies and doctors’ offices to identify breaches. There should be a dedicated cadre of trained personnel to enforce the law and prosecute offenders.
3. Trade and Industry- Standardization of drug prices across the Caribbean may reduce the large differential which facilitates this trade. Multilateral organizations such as the Caribbean Community (CARICOM) with its harmonized trade regimen must play a greater role in the exercise of its trade regulatory functions. This would allow greater bargaining and advocacy power in the international political economy.
4. Finance- Governments to purchase drugs which, although not profitable, are required in small volumes in the market. This would allow the needs of vulnerable populations to be met, ensuring the provision of the required therapeutics.
5. Health- Drug registration processes need to be more efficient. Health ministries should continue to be the centralized governance overseer for pharmaceuticals. An expanded list of approved drugs would foster competition and is likely to drive down prices.
6. Information Technology- The recognition of prescribing, utilization and supply chains of pharmaceuticals should be driven and empowered by information technology. This allows the governing structures to have awareness of gaps and potential breaches.
7. National Security- With its suite of law enforcement and detection powers, there is need for scanning and detection equipment at points of entry and borders and greater on-the- spot capacity to conduct quality assurance of medication.

## Conclusion

The examples of Ivermectin and HCQ during the pandemic are two of many in the informal and unregulated suitcase trade. The trade has the potential to cause serious harm to population health and as such requires a collaborative approach to mitigation. There must be sensitization of policy makers to the root causes of the problem and the necessity for multisectoral collaboration for mitigation. The over focus on third-party certification, verification and paper compliance without local capacity building, both in terms of equipment and human resource can do little to arrest the scourge. The authors believe that this is an underexplored topic that would benefit from further research involving pharmacists, the public and other stakeholders. It is only when this collaborative multisectoral approach is meaningfully undertaken that the deleterious effects of the suitcase trade in pharmaceuticals can be mitigated.

## Data Availability

The original contributions presented in the study are included in the article/supplementary material, further inquiries can be directed to the corresponding author.
